# Commentary: Long-Term Changes of Inflammatory Biomarkers in Individuals on Suppressive Three-Drug or Two-Drug Antiretroviral Regimens

**DOI:** 10.3389/fimmu.2022.904689

**Published:** 2022-04-29

**Authors:** Josep M. Llibre, Pedro E. Cahn, Tristan J. Barber

**Affiliations:** ^1^Infectious Diseases Division, University Hospital Germans Trias, Barcelona, Spain; ^2^Fight AIDS and Infectious Diseases Foundation, Barcelona, Spain; ^3^Fundación Huésped, Buenos Aires, Argentina; ^4^Ian Charleson Day Centre, Royal Free London NHS Foundation Trust, London, United Kingdom; ^5^Institute for Global Health, University College London, London, United Kingdom

**Keywords:** biomarkers, IL-6, sCD14, inflammation, d-dimer, C-reactive protein, antiretroviral therapy, dolutegravir/lamivudine

We read the recent article by Serrano-Villar et al. with great interest (1). The authors concluded that in virally suppressed people living with HIV (PLWH), maintaining a three-drug regimen (3DR) was associated with a more favorable long-term inflammatory profile than switching to two-drug regimens (2DR).

As this conclusion could have potential clinical implications, we believe that it is important to raise some concerns.

The authors included many different 3DR and 2DR, with a range of boosted protease inhibitors (lopinavir, atazanavir, and darunavir) and integrase inhibitors (cobicistat-boosted elvitegravir, dolutegravir, and raltegravir). As there is no evidence that all these different 3DR or 2DR regimens would have the same impact on inflammatory biomarkers among them, methodologically this merging is wrong.

The 2DR most frequently used today is dolutegravir/lamivudine, listed as preferred initial and switch therapy in all antiretroviral treatment guidelines (2–4). Dolutegravir/rilpivirine is also considered a potential regimen in switch. The study sample includes only 7 and 36 participants with these regimens, respectively, and is not powered for any conclusion regarding dolutegravir-based 2DR in contemporary use.

PLWH who switched to 2DR, particularly in the early period analyzed in the study (prescribed at 2005–2009) when 2DR was not commonly used, usually had a clinical reason to do so. These included drug–drug interactions, polypharmacy, comorbidities, or high cardiovascular risk, which are not necessarily captured in the variables collected. This makes it unlikely that both populations were comparable, even if the available variables were adjusted for.

Doing these analyses in cohorts entail a high risk of uncertainty. Inflammatory biomarkers may be impacted by smoking, obesity, type 2 diabetes, chronic obstructive lung disease, hazardous alcohol consumption, active chronic hepatitis, illicit drug use, depression, menopause, and other lifestyle factors (5). Thus, many putative drivers of inflammatory state in PLWH may not be corrected with plasma HIV suppression and may be independent of the chosen antiretrovirals. Once HIV suppression has been achieved and maintained, it is highly unlikely that different dolutegravir- or bictegravir-based antiretroviral regimens could be associated with significantly different levels of inflammation or immune activation unless they include drugs with intrinsic toxicity. Actually, a similar CD4/CD8 ratio recovery has been reported after initiation of a 2DR (dolutegravir plus lamivudine) versus dolutegravir- or bictegravir-based 3DR in naive PLWH (6).

Most markers of inflammation (e.g., interleukin 6 [IL-6], soluble tumor necrosis factor receptor 1, and high-sensitivity C-reactive protein [hs-CRP]), monocyte/macrophage activation (soluble CD14 [sCD14]), gut epithelial barrier dysfunction (fatty acid binding protein-2 [FABP-2]), and many others predict increased mortality during treated HIV infection, even among those with high current CD4+ T-cell counts (5).

Interpretation of these sensitive and unspecific biomarkers must be done with extreme caution and a high concordance among different biomarkers impacted by the same underlying mechanism (i.e., persistent inflammation) must be demanded for consistency. Doing these analyses in a small, non-randomized population can be prone to error.

Randomized studies have demonstrated the noninferiority of dolutegravir/lamivudine in rates of plasma HIV-RNA below 50 copies/ml up to 144 weeks with no consistent pattern of change in inflammatory markers (7–9). Exploratory analyses of rates of blips and target not detected plasma HIV-RNA (any detectable HIV-RNA below 50 copies/ml) consistently confirm similar rates of ultra-virologic suppression below the standard 50 copies/ml threshold, in both initial and switch therapy, up to 144 weeks (8, 10, 11), and also with dolutegravir/rilpivirine in switch (12, 13).

With so many biomarkers and endpoints analyzed and potential laboratory issues with different sample batches, it is not surprising that some significant differences are seen. Both SALSA and TANGO studies with dolutegravir/lamivudine and SWORD with dolutegravir/rilpivirine have shown a significant improvement of sCD14 at 48 and 144 weeks, a marker of monocyte activation released upon inflammatory cytokines (8, 9). IL-6 worsened in TANGO but not in SALSA at 48 weeks (8). Similarly, an improvement in FABP-2 and sVCAM-1 in addition to sCD14, with a worsening in IL-6 and sCD163, has been seen in SWORD with dolutegravir/rilpivirine at 148 weeks, though all study subjects were receiving dolutegravir/rilpivirine since week 52 (14).

Serrano-Villar et al. claim that the lack of correlation between biomarkers that should consistently be impacted in the same way indicates that each one represents an independent biological pathway, and suggests a direct impact of the 2DR vs. 3DR regimens (1). They state that the IL-6 increases with 2DR as observed in TANGO, SWORD, and also in their observational study (*n* = 7) support the hypothesis that 3DR might exert stronger anti-inflammatory effects. We disagree, as IL-6 increases were not seen at 48 weeks in SWORD with dolutegravir/rilpivirine, and at 148 weeks, such increases were inconsistently seen in only one of the four study groups (12, 14). The authors consider the most likely mechanism to be related to the magnitude of HIV RNA expression and translation in locations where drugs are poorly distributed, mainly lymphoid tissue. In our opinion, there is no evidence to support this statement. In fact, a strong correlation between biomarkers impacted by the same condition, namely, IL-6, sCD14, hs-CRP, and D-dimer, has generally been shown in most cohort studies, and all of them were predictors of AIDS, serious non-AIDS events, mortality, and a broad array of morbidities (5, 15–18). While a different mechanistic pathway may underlie the intrinsic activation of every biomarker, with such uncertainty even in randomized studies with the highest level of accuracy, the interpretation of isolated non-concordant results must be extremely cautious, even if statistical significance in any direction is identified with a given biomarker and timepoint.

We have thoroughly reviewed the changes in inflammatory and atherogenesis biomarkers with dolutegravir/lamivudine in treatment-experienced, virologically suppressed PLWH in a systematic literature review and found no evidence to support a difference with a comparator 3DR (19). Our interpretation has always been that extreme caution is needed in the interpretation of these results, and that the evidence so far suggests similar changes in inflammatory biomarkers.

Finally, Serrano-Villar et al. considered that it would be important to analyze the impact of these differences on the risk of clinical events in very large studies. The rates of clinical outcomes have already been analyzed in 1,088 PLWH on 2DRs and 8,703 on 3DRs in the RESPOND European-Australian consortium with 619 events during 27,159 person-years of follow-up (20). There was a similar incidence of clinical events on 2DRs and 3DRs in all events, validated events, or even in an adjusted analysis restricted to only guideline-recommended regimens [incidence rate ratio 1.28 (95% CI 0.88–1.87)].

In summary, there is compelling evidence that dolutegravir/lamivudine or dolutegravir/rilpivirine has a comparable impact on inflammatory and atherogenesis biomarkers versus 3DRs in their pivotal randomized controlled trials. There is currently no scientific proof of a differential impact of a dolutegravir-based 2DR on inflammatory biomarkers or any evidence of worse clinical outcomes compared to 3DR.

## Author Contributions

JL conceptualized the analysis and the manuscript. PC and TB were involved in the drafting and review of the manuscript and approved the final version. All authors contributed to the article and approved the submitted version.

## Conflict of Interest

JL has received payments or honoraria for lectures, presentations, or speaker’s bureaus from Janssen-Cilag, Gilead Sciences, and ViiV Healthcare, outside of the present work. PC has received research grants from Richmond and ViiV Healthcare, consulting fees from ViiV Healthcare; has received honoraria for speaker fees from ViiV Healthcare and Novartis; and has participated in data safety monitoring/advisory boards for Moderna, ViiV Healthcare, and Merck. TB has received conference support, speaker fees, and advisory board honoraria from Gilead Sciences, Janssen, MSD, Roche, Theratech, and ViiV Healthcare, and holds research and educational grants from Gilead Sciences, Roche, and ViiV Healthcare.

## Publisher’s Note

All claims expressed in this article are solely those of the authors and do not necessarily represent those of their affiliated organizations, or those of the publisher, the editors and the reviewers. Any product that may be evaluated in this article, or claim that may be made by its manufacturer, is not guaranteed or endorsed by the publisher.
